# Usefulness of the MRP2 promoter to overcome the chemoresistance of gastrointestinal and liver tumors by enhancing the expression of the drug transporter OATP1B1

**DOI:** 10.18632/oncotarget.16119

**Published:** 2017-03-11

**Authors:** Elisa Herraez, Laura Sanchez-Vicente, Rocio I.R. Macias, Oscar Briz, J.G. Marin Jose

**Affiliations:** ^1^ Experimental Hepatology and Drug Targeting (HEVEFARM), IBSAL, University of Salamanca, Salamanca, Spain; ^2^ Center for the Study of Liver and Gastrointestinal Diseases (CIBERehd), Carlos III National Institute of Health, Madrid, Spain

**Keywords:** ABC proteins, colon cancer, gene therapy, drug targeting, drug transporter

## Abstract

Tumor response to chemotherapy is often limited by drug export through ABC proteins. To overcome this problem, here we have investigated the usefulness of inducing the expression of the multidrug uptake transporter OATP1B1 under the control of the MRP2 promoter (MRP2pr). Human hepatoma cells (Alexander) were transfected with MRP2pr fragments of different length fused to the firefly luciferase ORF in order to select the shortest fragment with the highest response to dexamethasone, which was subsequently used to generate the chimeric construct MRP2pr-OATP1B1-V5. Hepatoma cells transduced with MRP2pr-OATP1B1-V5 resulted in dexamethasone-sensitive inducible OATP1B1 expression and enhanced selective antitumor response to OATP1B1 substrates (paclitaxel, Bamet-R2 and Bamet-UD2). In human colon cancer cells LS174T/R, used as a model of endogenous chemoresistance due to MRP2 overexpression, MRP2pr-OATP1B1 induced OATP1B1 expression together with chemosensitivity to OATP1B1 substrates. In nude mice, xenografted tumors formed by LS174T/R cells transduced with MRP2pr-OATP1B1 plus treatment with dexamethasone were markedly sensitized to Bamet-UD2. In conclusion, the induced expression of anticancer drug uptake transporters, under the control of promoters of ABC proteins involved in chemoresistance, constitutes an interesting approach to overcome the poor response of cancer to chemotherapy due to reduced drug uptake and/or enhanced drug export.

## INTRODUCTION

Primary malignancies of the liver and the gastrointestinal tract constitute one of the most common causes of death due to cancer worldwide [1]. Although surgery is the curative therapy of choice, the resection of these tumors is not always possible, and treatment often requires the use of alternative therapies. Systemic chemotherapy is the most common option for treating advanced disease [2]. Unfortunately, despite the long list of drugs available for the treatment of these tumors, the efficacy of pharmacological approaches is very poor due to the pre-existence of refractoriness to antitumor drugs and/or the development of chemoresistance during treatment.

Mechanisms of chemoresistance (MOC) are present in both cancer cells and healthy tissues, where they are involved in the defense against the chemical stress caused by potentially toxic compounds [3]. A group of MOC, classified as MOC-1b, accounts for the reduction of intracellular concentrations of the active agent by its efficient export across the plasma membrane [3]. Several members of the superfamily of ATP-binding cassette (ABC) proteins play an important role in the multidrug-resistance (MDR) phenotype, because they are able to export a large variety of drugs [4, 5]. Most of these transporters, such as the multidrug resistance-associated protein-2 (MRP2, gene symbol *ABCC2*) are highly expressed in tumor cells. The overexpression of MRP2 is considered an important mechanism accounting for the failure of many antitumor drugs commonly used in the treatment of liver and gastrointestinal cancers [6, 7].

Under physiological conditions, MRP2 is located at the apical membrane of several types of polarized cells, where this pump plays a key role in the efflux and detoxification of endogenous and xenobiotic toxic compounds [8]. Thus, MRP2 is involved in the elimination from hepatocytes into bile, from kidney proximal tubule epithelial cells into urine, and from intestinal epithelial cells into the intestinal lumen, of a large variety of molecules, which are for the most part conjugated with glutathione, sulfate or glucuronic acid [9–11]. Regarding antitumor drugs, MRP2 is able to export anthracyclines, camptothecins, chlorambucil, cisplatin, cyclophosphamide, methotrexate, podophyllotoxins, tamoxifen, and Vinca alkaloids [6, 12–18]. Interestingly, MRP2 is up-regulated during treatment with anticancer agents, some of which, but not all, are substrates of this pump [19, 20]. The direct consequence is an increase in the ability of these cells to export these drugs, which reduces their intracellular accumulation, resulting in the development of resistance, often cross-resistance, of tumor cells to chemotherapy [8, 21].

Reduced therapeutic efficacy of anticancer drugs can also be due to changes in the expression and/or activity of the transporters involved in drug uptake, which have been classified as MOC-1a [3]. The most important plasma membrane carriers involved in drug uptake by the liver and gastrointestinal tissues belong to the family of solute carrier (SLC) proteins [20]. In neoplastic tissues the expression and/or function of these proteins can be reduced during tumor development and/or under anticancer drug pressure. The consequence is lower intracellular drug concentrations and hence reduced antitumor activity of these agents in cancer cells. One of these transporters is OATP1B1 (gene symbol *SLCO1B1*), which plays an important role in the hepatic uptake of anionic antitumor drugs, such as irinotecan [22], paclitaxel [23] and cytostatic cisplatin-conjugated bile acid derivatives [24]. Interestingly, the OATP1B1 expression is decreased in primary liver cancer [21, 25].

Novel approaches are currently being developed to overcome chemoresistance in liver and gastrointestinal tumors, especially as this is mainly due to MOC-1a and/or MOC-1b. These include strategies already used in clinical practice that involve new drugs with an enhanced selectivity toward molecular targets, such as inhibitors of receptors with tyrosine kinase activity (TKIs) or with organ tropism, such as bile acid-conjugated derivatives. Another approach, the one being considered in the present study, is the chemosensitization of cancer cells by enhancing the intracellular concentration of the active drug through changes in the expression of uptake transporters. More specifically, as a proof-of-concept, we have evaluated the possibility of using gene therapy based on a chimeric construct containing the ORF of OATP1B1 under the control of the MRP2 promoter (MRP2pr). The objective was to chemosensitize tumor cells, with a reduced expression of OATP1B1 (MOC-1a) and a high expression of MRP2 (MOC-1b), to antitumor drugs efficiently transported by OATP1B1. The potency of this strategy can be further enhanced by combination with agents able to activate MRP2pr, such as dexamethasone [26, 27]. Previous attempts to favor drug uptake in cancer cells have been done. For instance, with diagnostic and therapeutic purposes, gene therapy based on the sodium/iodide symporter (NIS) gene (SLC5A5) has been investigated [28]. The aim of this approach was to induce NIS expression and hence increase radiolabeled iodide accumulation in cancer cells [28]. This has been assayed in several types of cancer including hepatocellular carcinoma [29]. Tissue specificity has been further improved by using promoters of cell-specific genes such as alpha-fetoprotein in hepatocellular carcinoma [29], prostate-specific membrane antigen in prostate cancer [30], and glial fibrillary acidic protein in glioma [31].

## RESULTS

### Selection of the MRP2 promoter fragment

**Figure 1:**
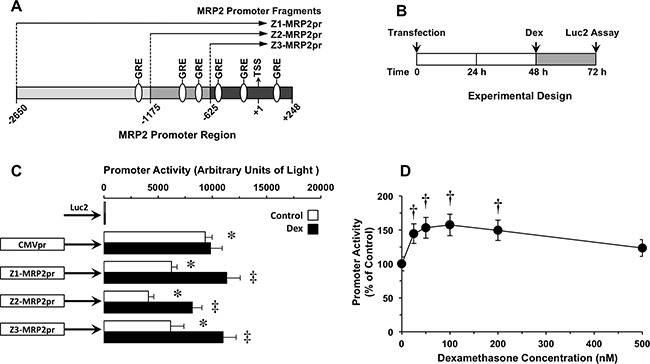
(A) Schematic representation of three (Z1, Z2 and Z3) 5’-located partial fragments of the MRP2 promoter (MRP2pr) that were cloned into plasmids containing the firefly luciferase gene (Luc2) Putative glucocorticoid response elements (GRE), potentially involved in the regulation of MRP2 expression are depicted. Numbers indicate positions relative to the main transcription start site (TSS) (+1). **(B)** Scheme of the experimental design used to determine the effect of dexamethasone (Dex) on MRP2pr activity in human hepatoma Alexander cells transiently transfected with Z1-, Z2 or Z3-MRP2pr-Luc2 plasmids. **(C)** Effect of Dex (100 nM) on Z1-, Z2- or Z3-MRP2pr transcriptional activity. Luc2 expression under the control of either cytomegalovirus promoter (CMVpr) or no promoter was used as positive and negative controls, respectively. **(D)** Dose-dependent effect of Dex in cells expressing Z1-MRP2pr-Luc2. Values are mean±SD from at least 3 transfection experiments performed in triplicate. †, p<0.05, compared to untreated cells (Control) by paired t-test. Bonferroni method of multiple range testing was used to compare to Luc2 in the absence of promoter (*, p<0.05) or on comparing with and without Dex treatment (‡, p<0.05).

In order to identify the region of the MRP2pr with the shortest size but the highest activating ability, several fragments (Z1-, Z2- and Z3-MRP2pr) of different length located 5’-upstream of the MRP2 transcription start site were investigated. In addition, we determined their ability to respond to the well-known MRP2 inductor dexamethasone, which mediates the induction of the transcription process through the activation of glucocorticoid response elements (GRE) previously identified in the MRP2pr (Figure 1A) [26, 27]. The MRP2pr fragments were previously cloned into plasmids to drive the expression of 3’-downstream located firefly luciferase (Luc2) [19]. Transfection of Alexander cells with vectors containing MRP2pr fragments fused to Luc2 and their treatment with dexamethasone were carried out as indicated in Figure 1B. Transfection of Alexander cells with a similar but promoter-less construct showed poor luciferase activity (Figure 1C). In contrast, all MRP2pr fragments markedly stimulated the luciferase activity. The efficacy was different among them and lower than that of the positive control cytomegalovirus promoter (CMVpr)-Luc2. The order of transcriptional activity was CMV>Z3≈Z1>Z2 (Figure 1C). Dexamethasone stimulated Z1-MRP2pr activity in a concentration-dependent manner (Figure 1D). Based on these results, 100 nM dexamethasone was used in further *in vitro* studies on MRP2pr activation by this glucocorticoid. The fragments of MRP2pr that preceded Luc2 showed different ability to respond to dexamethasone-induced luciferase activity (Figure 1C). Based on the results obtained in these experiments, Z3-MRP2pr was selected as the shortest fragment with the highest promoter activity. Accordingly, for the sake of clarity, Z3-MRP2pr will hereinafter be referred to as simply MRP2pr.

### OATP1B1 expression under the control of MRP2 promoter

**Figure 2:**
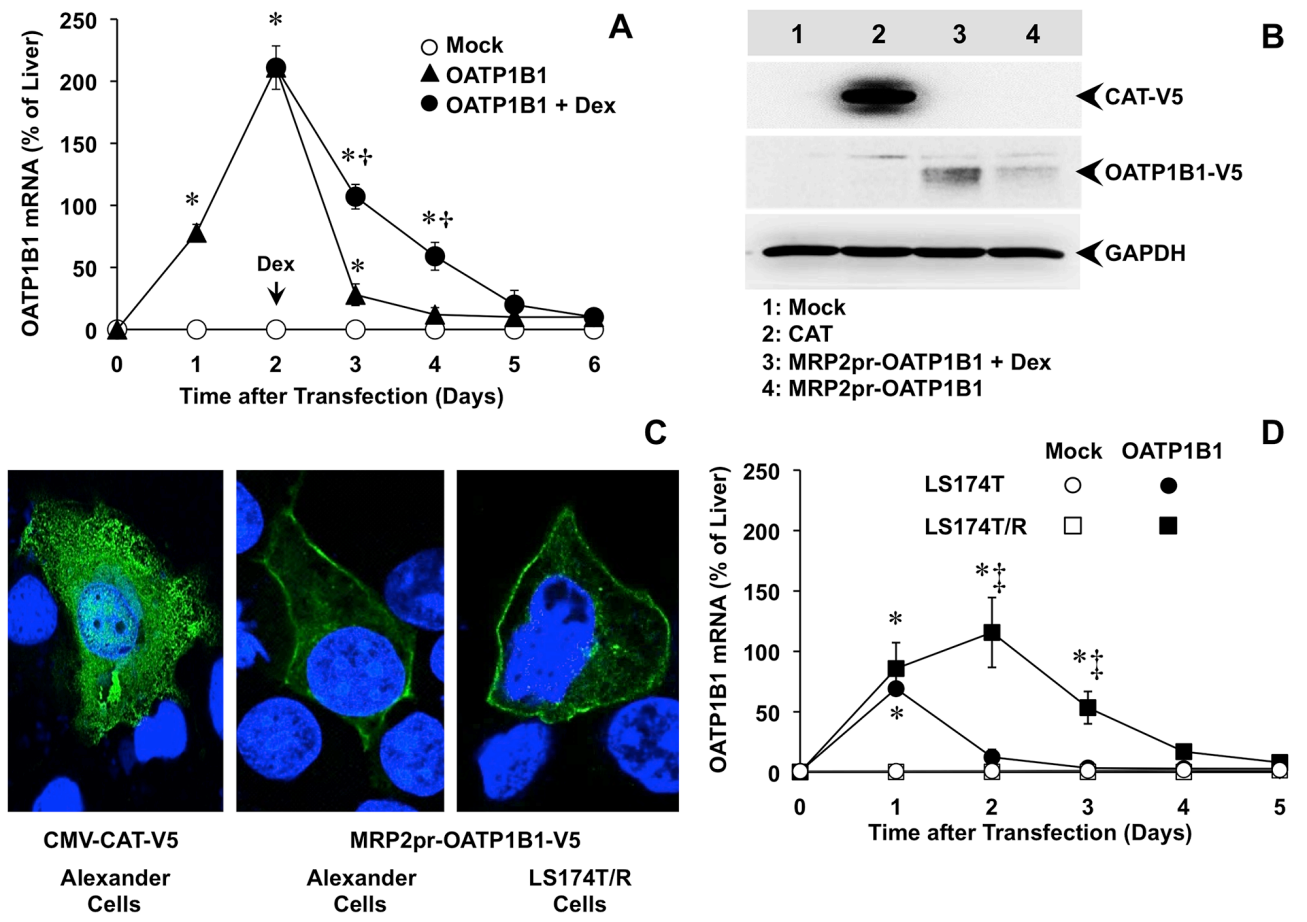
**(A)** Time-course of OATP1B1 expression in Alexander cells after transfection with a plasmid containing Z3-MRP2pr-OATP1B1-V5 or an empty vector (Mock). 48 h after transfection, OATP1B1 expressing cells were treated with 100 nM dexamethasone (Dex) or the vehicle. **(B)** Representative Western blot of OATP1B1-V5 in lysates from Alexander cells transfected with Z3-MRP2pr-OATP1B1-V5, treated with 100 nM Dex or the vehicle for 24 h. As a positive control of V5 expression a plasmid containing the V5-tagged ORF of chloramphenicol acetyl transferase (CAT) under the control of CMV promoter was used. An empty vector (Mock) was used as a negative control. Western blots were carried out with an antibody against the V5 epitope. GAPDH was used as loading protein normalizer in each lane. **(C)** Representative fluorescence confocal microscopy pictures of Alexander and LS174T/R cells expressing V5-tagged OATP1B1 or CAT, which were detected by using a monoclonal antibody against the V5 epitope (green). Nuclei were counterstained with DAPI (blue). **(D)** Time-course of OATP1B1 expression in human LS174T cells from colorectal adenocarcinoma and in the chemoresistant subline LS174T/R after transfection with a plasmid containing Z3-MRP2pr-OATP1B1-V5 or an empty vector (Mock). The amount of mRNA was determined by RT-QPCR and expressed as a percentage of that found in human liver. Values are expressed as mean±SD from 3 independent experiments performed in triplicate. *, p<0.05, compared to Mock cells; †, p<0.05, on comparing with and without Dex treatment. ‡, p<0.05, on comparing LS174T and chemoresistant LS174T/R cells. The Bonferroni method of multiple range testing was used for comparisons among groups.

Subsequent experiments were carried out to induce overexpression of OATP1B1, whose down-regulation in liver cancer cells constitutes an important limitation for the uptake and effectiveness of several antitumor drugs [22, 23]. Plasmids carrying the OATP1B1 ORF under the control of MRP2pr were transiently transfected into Alexander cells, which have negligible endogenous expression of OATP1B1. To easily follow the expression and subcellular localization of this protein, the V5 epitope was included in the 5’-end of the construct before the stop codon. After transfection, OATP1B1 mRNA levels increased and peaked at 48 h (Figure 2A). Afterward, mRNA levels progressively decreased and returned to negligible basal values at approximately 5 days after transfection. Treatment with dexamethasone (100 nM, from 48 h) significantly increased OATP1B1 expression, which remained during 4 days after transfection at levels higher than 50% of those detected in the control liver (Figure 2A). Western blot analysis confirmed that the increased expression resulted in higher abundance of OATP1B1 protein. This was further enhanced by treatment with dexamethasone. Alexander cells transfected with a CMV-CAT-V5 plasmid were used as a positive control (Figure 2B). Immunofluorescence analysis revealed that the detection of V5 in cells transfected with CMV-CAT-V5 plasmid was intracellular whereas when V5 was fused to OATP1B1 most of the fluorescent signal was located at the plasma membrane (Figure 2C).

To investigate the ability of MPR2pr activation to enhance OATP1B1 expression in an *in vitro* model of tumor cells with endogenous chemoresistance, LS174T cells derived from human colorectal adenocarcinoma and its derived chemoresistant subline LS174T/R were transiently transfected with the construct. This transfection induced moderate OATP1B1 expression in LS174T cells, consistent with low MRP2 expression in these cells [19]. In contrast, OATP1B1 was highly expressed in LS174T/R cells (Figure 2D), which are characterized by high MRP2 expression [19]. Similar to that described above for Alexander cells, the highest levels of OATP1B1 mRNA in LS174T/R cells were detected 48 h after transfection. In these cells, OATP1B1 protein was also localized at the plasma membrane (Figure 2C).

### OATP1B1 expression induced enhanced sensitivity to anticancer drugs

**Figure 3:**
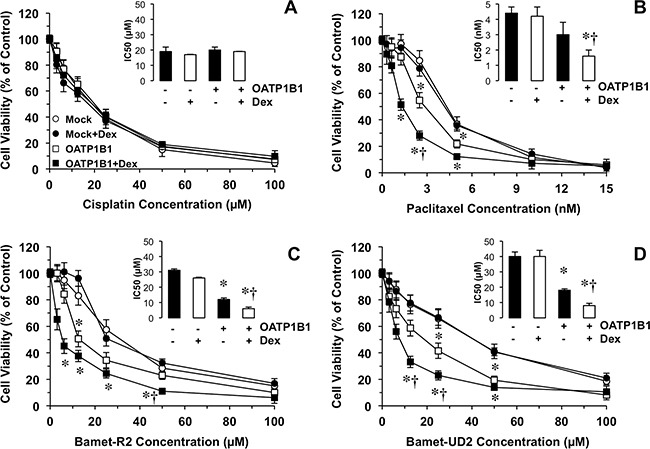
Antiproliferative effect of cisplatin **(A)** and three OATP1B1 substrates. Paclitaxel **(B)** and the cisplatin-bile acid conjugates Bamet-R2 **(C)** and Bamet-UD2 **(D)**, were used to treat human hepatoma Alexander cells transfected with a plasmid containing Z3-MRP2pr-OATP1B1-V5 or empty vector (Mock). After transfection (48 h), cells were incubated with anticancer agents with or without 100 nM dexamethasone (Dex) for an additional 72 h period. Insets: Inhibitory concentration 50 (IC50) of cisplatin, paclitaxel, Bamet-R2 and Bamet-UD2, in Alexander cells transfected with a plasmid containing Z3-MRP2pr-OATP1B1-V5 (+) or empty vector (-) and treated with or without Dex. Values are expressed as mean±SD from 3 independent experiments performed in triplicate. *, p<0.05, on comparing to Mock cells. †, p<0.05, on comparing to cells not treated with Dex. The Bonferroni method of multiple range testing was used for comparisons among groups.

**Figure 4:**
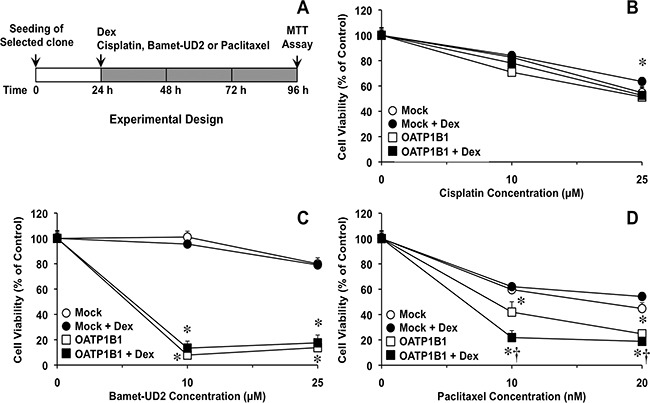
Scheme of the experimental design used to determine the effect of dexamethasone (Dex) on OATP1B1 expression-induced sensitivity to antitumor drugs **(A)**. Antiproliferative effect of cisplatin **(B)** Bamet-UD2 **(C)** and paclitaxel **(D)** in Alexander cells stably expressing OATP1B1 under the control of Z3-MRP2pr or transduced with a Mock lentiviral vector. Cells were incubated with the anticancer agents with or without 100 nM Dex for 72 h. Values are expressed as mean±SD from 3 independent experiments performed in triplicate. *, p<0.05 as compared with Mock cells. †, p<0.05, on comparing with and without Dex treatment. The Bonferroni method of multiple range testing was used for comparisons among groups.

To evaluate the ability of the chimeric construct to sensitize cancer cells to cytostatic OATP1B1 substrates, a dose-dependent study was carried out in Alexander cells incubated with the desired antitumor drug from 48 h after the transiently transfection (Figure 3), based on the results obtained in the time-course of OATP1B1 expression described above (Figure 2A). OATP1B1 expression had no effect on the sensitivity of Alexander cells to cisplatin, (Figure 3A), which is not transported by this carrier. In contrast, the cytotoxic effect of paclitaxel (Figure 3B) and two cytostatic bile acid derivatives, i.e., Bamet-R2 (Figure 3C) and Bamet-UD2 (Figure 3D), was enhanced by OATP1B1 expression. The sensitivity to these drugs was further increased by treatment with dexamethasone. Thus, under these conditions, a marked reduction in IC50 values for paclitaxel (2.7-fold), Bamet-R2 and Bamet-UD2 (both ˜5-fold) was found (Figure 3, insets). In contrast, the antitumor effect of these drugs was not enhanced by dexamethasone in cells that were transfected with the empty vector (Mock). The chemosensitizing ability of this strategy was confirmed in an *in vitro* model of stable overexpression of OATP1B1 using lentiviral vectors (Figure 4A). In stably transduced cells, neither OATP1B1 expression nor dexamethasone treatment affected the sensitivity to cisplatin (Figure 4B). In contrast, the maintained overexpression of OATP1B1 was able to induce enhanced sensitivity of Alexander cells to Bamet-UD2 (Figure 4C) and paclitaxel (Figure 4D). The effect of Bamet-UD2 in cells expressing OATP1B1 was so strong that there was no room for improvement by further increasing OATP1B1 by dexamethasone treatment. In contrast, although the effect of paclitaxel was improved by OATP1B1 expression, this could be further enhanced by dexamethasone-dependent OATP1B1 up-regulation.

**Figure 5:**
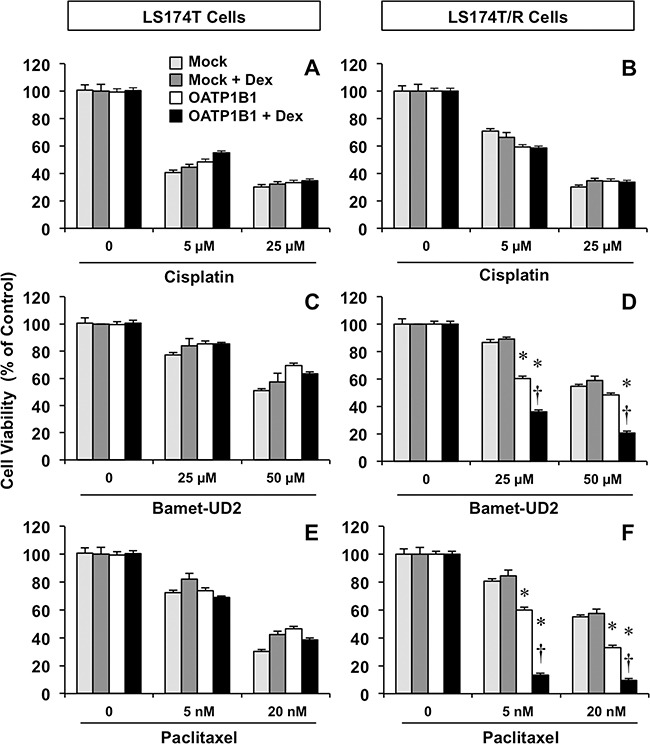
Antiproliferative effect of cisplatin **(A, B)** Bamet-UD2 **(C, D)** and paclitaxel **(E, F)** in LS174T cells (A, C, E) and the partially chemoresistant subline LS174T/R (B, D, F). Both cell types were transduced with lentiviral vectors, either empty (Mock) or containing Z3-MRP2pr-OATP1B1. Cells were incubated with anticancer agents with or without 100 nM dexamethasone (Dex) for 72 h. Values mean±SD from 3 independent experiments performed in triplicate are expressed as a percentage of untreated cells (Control). *, p<0.05 compared with Mock cells. †, p<0.05, on comparing with and without Dex treatment. The Bonferroni method of multiple range testing was used for comparisons among groups.

The ability of this chemosensitizing strategy to overcome endogenous chemoresistance was evaluated using paired wild-type (LS174T) and chemoresistant (LS174T/R) cells as a model of stable overexpression of OATP1B1 using lentiviral vectors (Figure 5). In chemosensitive LS174T cells, transduction with the chimeric construct was not associated with a higher sensitivity to cytostatic OATP1B1 substrates (Figure 5C and 5E). In LS174T/R cells, no difference between transduction with Mock and OATP1B1-bearing lentiviral vectors was found when these cells were treated with cisplatin alone or in combination with dexamethasone (Figure 5B). In contrast, stable expression of OATP1B1 increased the sensitivity of LS174T/R cells to Bamet-UD2 (Figure 5D) and paclitaxel (Figure 5F). This effect was enhanced by combined incubation with dexamethasone.

### Experiments in the mouse xenograft model

**Figure 6:**
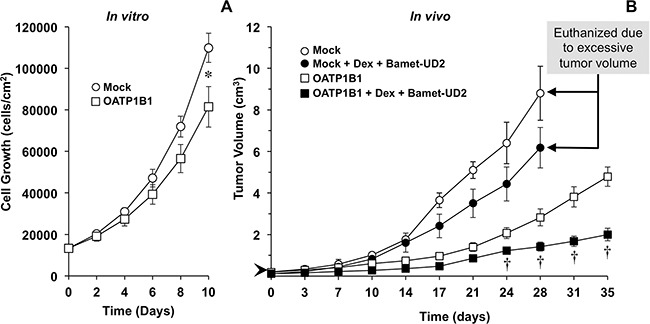
Time course of LS174T/R cells growth in culture *in vitro* **(A)** and as a tumor *in vivo* following subcutaneous injection in nude mice **(B)**. LS174T/R cells were transduced with an empty lentiviral vector (Mock) or a vector containing Z3-MRP2pr-OATP1B1. After implanting 1×10^7^ LS174T/R cells, nude mice were treated with Bamet-UD2 (15 nmol/g b.w., i.p.) and dexamethasone (Dex, 1.3 nmol/g b.w., i.p.) or the vehicle (saline) alone on days 0, 3, 7, 10, 14, 17, 21, 24, 28 and 31, starting when the tumors reached a size of ≈0.5 cm diameter (volume ≈0.06 cm^3^) (arrow head). Values are means±SD (n≥4 per group). *, p<0.05 as compared with cells overexpressing OATP1B1 by Student *t*-test; †, p<0.05 as compared with the appropriate untreated group by the Bonferroni method of multiple range testing.

We have previously shown the ability of Bamet-UD2 to inhibit the growth of tumors formed by LS174T cells *in vivo* [32]. Here we have evaluated the usefulness of chemosensitizing the chemoresistant subline LS174T/R to Bamet-UD2 by overexpressing OATP1B1 under the control of MRP2pr, which was further stimulated by treatment with dexamethasone. To reduce the number of animals to be used in this phase of the study, we have assayed only the strongest chemosensitizing strategy, based on results obtained from the *in vitro* phase. We have observed that the monoclonal subline of LS174T/R cells overexpressing OATP1B1 showed, on its own, slower growth rate *in vitro* (Figure 6A) and *in vivo* (Figure 6B) than the parental line. Whether mild tumor-suppressor effect of the Z3-MRP2pr-OATP1B1 construct itself was due to the insertion of the gene in an interfering site of the genome or to enhanced sensitivity to inhibitory environmental factors is an interesting question that must be addressed in further investigations. In the absence of chemosensitization, Bamet-UD2 had only a moderate effect on tumor growth. In contrast, in tumors overexpressing OATP1B1, the antitumor effect of Bamet-UD2 was significantly enhanced (Figure 6B).

## DISCUSSION

The limited success of available treatments for gastrointestinal and liver cancer makes the development of more efficient therapeutic approaches both necessary and urgent. Gene therapy has emerged as an alternative to conventional treatments. One of the major obstacles for the use of gene therapy is the specific targeting of transgene expression to the site of the tumor. Numerous gene therapy strategies have used nonspecific and nonselective promoters that can be expressed at high levels even in healthy cells, potentially contributing to toxicity. For cancer treatment a better strategy would be the use of tissue and/or cancer-specific promoters to limit the expression of therapeutic genes in the desired target cells [33, 34].

Under physiological circumstances, MRP2 is normally expressed in liver, intestine and kidney [35–37], however this pump is frequently overexpressed in tumor cells derived from these and other tissues. MRP2 expression is further stimulated in response to many structurally different chemotherapeutic agents. Thus, the MOC-1b accounted for by MRP2-mediated drug efflux is considered an important cause of the poor response to anticancer chemotherapy [7, 21, 38]. Based on this common tumor characteristic, the use of the MRP2pr emerged as a good option for enhancing the efficacy of gene therapy in these tumors by combining its strong transcriptional activity with a high degree of specificity. Moreover, this promoter has two additional advantages: it is inducible by conventional cancer agents [19] and it could be further pharmacologically activated, for instance by co-treatment with dexamethasone.

An important limitation of tissue or cancer-specific promoters is that most of them are much weaker than commonly used viral promoters such as the SV40 promoter or the CMV promoter. The results obtained in our study regarding the activity of different fragments of the MRP2pr showed that, although under basal conditions their activity in human hepatoma cells was lower than those observed for the constitutive CMV promoter, they present, in contrast to CMV, the ability to respond to pharmacological activation, for instance with dexamethasone. Z3-MRP2pr was the fragment of MRP2pr that showed the strongest response to dexamethasone, probably because it still contained the majority of GRE, and lacks inhibitory response elements present in longer fragments. The selection of this fragment, markedly shorter than the complete promoter sequence, to drive the expression of the therapeutic gene will constitute an advantage in further steps of the development of this strategy, because size limitation is a common problem in the design of viral vectors with limited cargo capability, such as adeno-associated viral vectors. As shown by our results, the fragment Z3-MRP2pr was efficient in driving OATP1B1 expression. Moreover, this protein was correctly targeted at the plasma membrane of hepatoma cells, resulting in enhanced selective sensitivity to antitumor OATP1B1 substrates, such as paclitaxel and cisplatin-bile acid conjugates (Bamet-R2 and Bamet-UD2). This has important pharmacological implications because the frequent loss of OATP1B1 expression in liver cancer [21, 25] markedly limits the antitumor drug uptake [22, 23].

Although ABCC2 is poorly expressed in healthy colorectal epithelium [39], the expression of this pump is significantly elevated in colorectal adenocarcinoma [7]. This partly accounts for the high resistance to platinum derivatives currently used for the treatment of these tumors in combination with other drugs [40, 41]. The experiments performed here using human colon cancer cells have demonstrated that our therapeutic construct is able to induce higher sensitivity to OATP1B1 antitumor substrates, which occurs specifically in colon cancer cells that overexpress MRP2. Moreover, further activation of MRP2pr by dexamethasone dramatically increased the sensitivity of the chemoresistant cells to paclitaxel and cytostatic bile acid derivatives.

Treatment of colon cancer cells with cisplatin stimulates the development of MDR phenotype, which is characterized by the up-regulation of several ABC proteins, in particular MRP2 [19]. This supports the hypothesis that gene therapy based on the strategy evaluated here may benefit patients that have developed drug resistance during treatment mainly due to enhanced drug export. Furthermore, the low expression of MRP2 in healthy colon tissue suggests that the activation of the MRP2pr will be selective for cancer cells and hence its use in gene therapy will not increase drug toxicity on healthy colon tissue. In other organs, such as liver and kidney, the up-regulation of OATP1B1 may favor drug uptake. Whether this may affect the pharmacokinetics of the drug or a selective enhancement in hepatotoxicity and nephrotoxicity are interesting questions that deserve further evaluation after this proof-of-concept study. Potential limitations and difficulties to use this strategy in clinic include the selection of appropriate vector and the need of approved antitumor drugs with substrate specificity for OATP1B1. Moreover, the success requires up-regulation of MRP2, thus in tumors overexpressing other ABC proteins, the promoter used in the construct should be selected after the identification of ABC profile in each individual case. Finally, it is possible that chemosensitized tumors develop alternative ways of chemoresistance during treatment, which would require dynamic adaptation to the evolution of the target tumor characteristics.

In conclusion, the enhanced expression of anticancer drug uptake transporters, such as OATP1B1, under the control of promoters of ABC proteins involved in chemoresistance, such as MRP2, constitutes an interesting approach to overcome the poor response to chemotherapy due to reduced drug uptake and enhanced drug export.

## MATERIALS AND METHODS

### Chemicals and cells

Cisplatin, dexamethasone and paclitaxel were obtained from Sigma-Aldrich (Madrid, Spain). According to the suppliers, the purity of these compounds was ≥97%. All other chemicals were of analytical grade. Synthesis, structure and chemical characteristics of bile acid derivatives Bamet-R2 and Bamet-UD2 (90% pure as determined by platinum absorption spectrometry) have been previously described [42, 43]. Alexander or PCL/PRF/5 (human hepatoma), LS174T (human colon adenocarcinoma) and HEK293T (human embryonic kidney) cells were obtained from the American Type Culture Collection (LGC Standards, Barcelona, Spain). Monoclonal chemoresistant LS174T/R cells overexpressing MRP2 were previously obtained and characterized as reported [19].

### OATP1B1 cloning into expression vectors

The human OATP1B1 ORF was amplified from total RNA isolated from healthy liver by reverse transcription followed by high-fidelity PCR using the AccuPrime *Pfx* DNA polymerase (Invitrogen, Thermo Fisher Scientific, Madrid) and specific primers containing the appropriate *attB* adapters (Supplementary Table 1). The *attB*-flanked PCR product was recombined with a pDONR221 vector (Invitrogen, Thermo Fisher Scientific) to generate a pEntry plasmid. This was simultaneously recombined with another pEntry plasmid containing one of the MRP2pr fragments previously obtained [19], and a promoter-less destination vector (pcDNA6.2-V5pL or pWPIpL-DEST) by Multisite Gateway® cloning (Invitrogen, Thermo Fisher Scientific) to obtain the desired expression vectors (Supplementary Figure 1). A promoter-less lentiviral destination vector (pWPIpL) was previously obtained by removing the EF1a promoter from the pWPI vector using high-fidelity PCR with the AccuPrime *Pfx* DNA polymerase and specific primers (Supplementary Table 1). The exact nucleotide sequence of all constructs was confirmed by gel-electrophoresis-based sequencing. Recombinant lentiviruses were produced in HEK293T cells transfected using a standard polyethylenimine (PEI) protocol with the transfer vector pWPI-Z3-MRP2pr-OATP1B1, encoding both OATP1B1 and the enhanced green fluorescent protein (EGFP) or simply pWPI (to generate “Mock vectors”), and the packaging plasmids psPAX2 and pMD2.G. Viral titers were determined by infection of HEK293T cells with serial dilutions of the viral solution, and the analysis of EGFP-positive cells was carried out 4 days later with a FACSCalibur flow cytometer (BD Biosciences, Madrid). Lentiviral vectors were added to target cells at a multiplicity of infection (MOI) of 5 infectious particles per cell in the presence of polybrene, and the infected cells were tested by flow cytometry (Supplementary Figure 2A) and by fluorescence microscopy (Supplementary Figures 2B and 2C). Monoclonal stably expressing sublines were obtained by using the limiting dilution method (Supplementary Figure 2D). The ability of infected cells to respond to dexamethasone was indirectly determined by analyzing, using flow cytometry, the fluorescence due to EGFP. The clone with the highest response to dexamethasone was selected for further studies (Supplementary Figures 2 and 3).

### Reporter gene assays

Alexander cells were transiently transfected with firefly luciferase (Luc2) fusion plasmids by using a lipofectamine LTX/PLUS reagent (Invitrogen), and cultured for 72 h before measuring promoter activation (Figure 1B), which was carried out by determining changes in luciferase activity. The expression of Luc2 was enzymatically determined by measuring the light generated in the presence of the substrate luciferin using the Bright-Glo Luciferase Assay System (Promega, Madrid) in a LAS-4000 image reader (FujiFilm TDI, Madrid). The results were expressed as relative units of light (arbitrary light units/min) corrected by the amount of living cells in each well. In some cases, 48 h after transfection, the cells were incubated with different concentrations of dexamethasone for 24 h before measuring luciferase activity (Figure 1B).

### Determination of OATP1B1 mRNA levels

DNA synthesized from total RNA by reverse transcription (RT) was used as a template to determine OATP1B1 expression by real-time quantitative PCR (QPCR) using gene-specific primers spanning the exon-exon junctions in the target mRNA (Supplementary Table 1) and AmpliTaq Gold DNA polymerase in a 7300 Real-Time PCR System (Applied Biosystems, Thermo Fisher Scientific). The thermal cycling conditions were as follows: single cycles at 50°C for 2 min and at 95°C for 10 min, followed by 40 cycles at 95°C for 15 s and at 60°C for 60 s. Detection of the amplification products was carried out using SYBR Green I. The mRNA abundance of OATP1B1 in each sample was normalized on the basis of its GAPDH content.

### Western blot

Immunoblotting analyses of cell lysates were carried out in 7.5% SDS-PAGE, loading 100 μg of protein per lane. Blots were probed with primary monoclonal antibodies against V5 (Invitrogen), and GAPDH (clone 6C5, Santa Cruz Biotechnology, Santa Cruz, CA). Horseradish peroxidase-linked chicken anti-mouse secondary antibody was from Invitrogen. An enhanced chemiluminescence detection system (Hybond ECL; GE Healthcare, Barcelona) was used to visualize the bands.

### Immunofluorescence analyses

Immunostaining was carried out in air-dried cells grown on coverslips, which were fixed and permeabilized with ice-cold methanol. The primary V5 antibody was diluted 1:800 in 5% fetal calf serum in PBS. The secondary Alexa Fluor-488 antibody (Life Technologies, Madrid) was diluted 1:1000. Nuclei were counterstained with 10 μM 4′,6-diamino-2-phenylindole (DAPI). Fluorescence staining was visualized using a Leica TCS SP2 confocal microscope.

### Cytostatic activity assays

To measure cytostatic activity *in vitro*, LS174T, LS174T/R cells (≈15000 cells/well) or Alexander cells (≈5000 cells/well) were seeded onto 96-well plates. In the case of cells transfected with pcDNA3.1-Hygro(+) or Z3-MRP2pr-OATP1B1-V5 plasmids, transfection was carried out 24 h after seeding and, 48 h later, the cells were incubated with the desired compound for 72 h. In the case of cell transduced with lentiviral vectors, 24 h after seeding, the cells were exposed to the desired compound in the presence or the absence of 100 nM dexamethasone for 72 h (Figure 4A). Cell viability was determined with the MTT test (Sigma).

### *In vivo* experiments

Eight weeks old female nude mice (Swiss *nu/nu*) were purchased from Charles River Laboratories (Barcelona) and were maintained under pathogen-free conditions and handled under stringent sterile conditions. The animals were fed on standard mouse chow (Panlab, Madrid) and water *ad libitum*. Temperature (20°C) and the light/dark cycle (12 h:12 h) were controlled and the protocols were approved by the Ethics Committee of the University of Salamanca. To study antitumor activity *in vivo*, nude mice were anesthetized with isoflurane and 1×10^7^ LS174T/R cells stably expressing OATP1B1 or transduced with pWPI vector (Mock), were suspended in 100 μl of Matrigel (BD Biosciences) and subcutaneously injected in the mouse flank. When the tumors reached a size of ≈0.5 cm in diameter (volume≈0.06 cm^3^), the animals were randomly divided into two groups that received Bamet-UD2 plus dexamethasone, or the vehicle alone (sterile saline) on days 0, 3, 7, 10, 14, 17, 21, 24, 28 and 31 after implanting the cells. The dose of Bamet-UD2 (15 nmol/g b.w., i.p.) was selected based on previous studies that demonstrated the antitumor effect of this compound *in vivo* without any detectable toxic side effect [44]. The selection of the dose of dexamethasone (1.3 nmol/g b.w., i.p.) was based on recent studies by our group on the dose-dependent hepatotoxicity of this glucocorticoid in mice [45]. Animals were monitored daily and tumors were measured twice per week with a sliding caliper to calculate the volume using the formula (length × width^2^)/2. The animals were euthanized by anesthesia overdose when the tumor volume reached an excessive volume that limited their movements and access to food and water.

### Statistical analyses

Data are presented as mean±SD. Using IBM SPSS Statistics V20 for Mac, paired or unpaired Student *t*-test, or the Bonferroni method of multiple-range testing were used as appropriate as post hoc procedure to calculate the statistical significance of differences among groups.

## SUPPLEMENTARY MATERIALS FIGURES AND TABLES



## Abbreviations

ABC: ATP binding cassette
CMV: cytomegalovirus
GRE: glucocorticoid response element
MDR: multidrug-resistance
MOC: mechanism of chemoresistance
MRP: multidrug resistance-associated protein
OATP: organic anion transporting polypeptide
SLC: solute carrier protein
