# Supplementary material for: Usefulness of the MRP2 promoter to overcome the chemoresistance of gastrointestinal and liver tumors by enhancing the expression of the drug transporter OATP1B1
Source: Oncotarget. 2017 Mar 11;8(21):34617–29. doi: 10.18632/oncotarget.16119 (PMC5470996; doi:10.18632/oncotarget.16119)
Supplement: Supplementary file 1 [file oncotarget-08-34617-s001.pdf]

Usefulness of the MRP2 promoter to overcome the chemoresistance of gastrointestinal and liver tumors by enhancing the expression of the drug transporter OATP1B1

SUPPLEMENTARY FIGURES AND TABLE

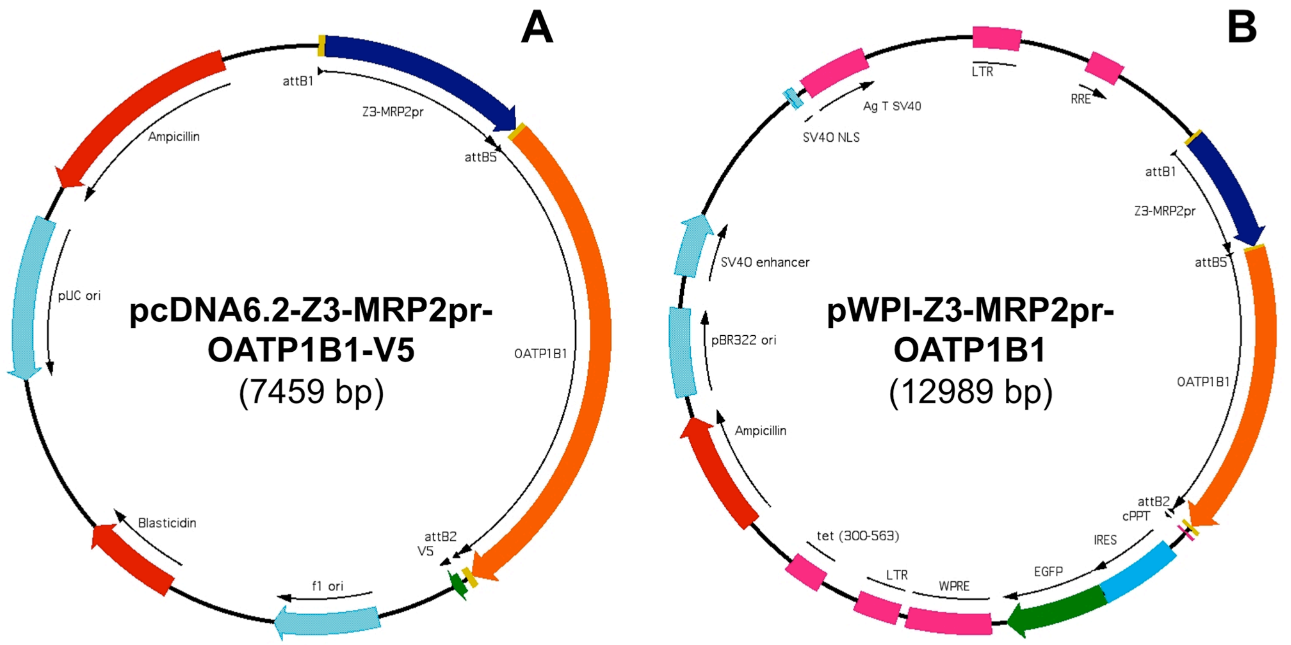

Supplementary Figure 1: Schematic representation of the vectors used for gene transduction of cultured cells by lipofectamine-based transfection using pcDNA6.2-Z3-MRP2pr-OATP1B1-V5 (A) or lentiviral-mediated transduction using pWPI-Z3-MRP2pr-OATP1B1 (B).

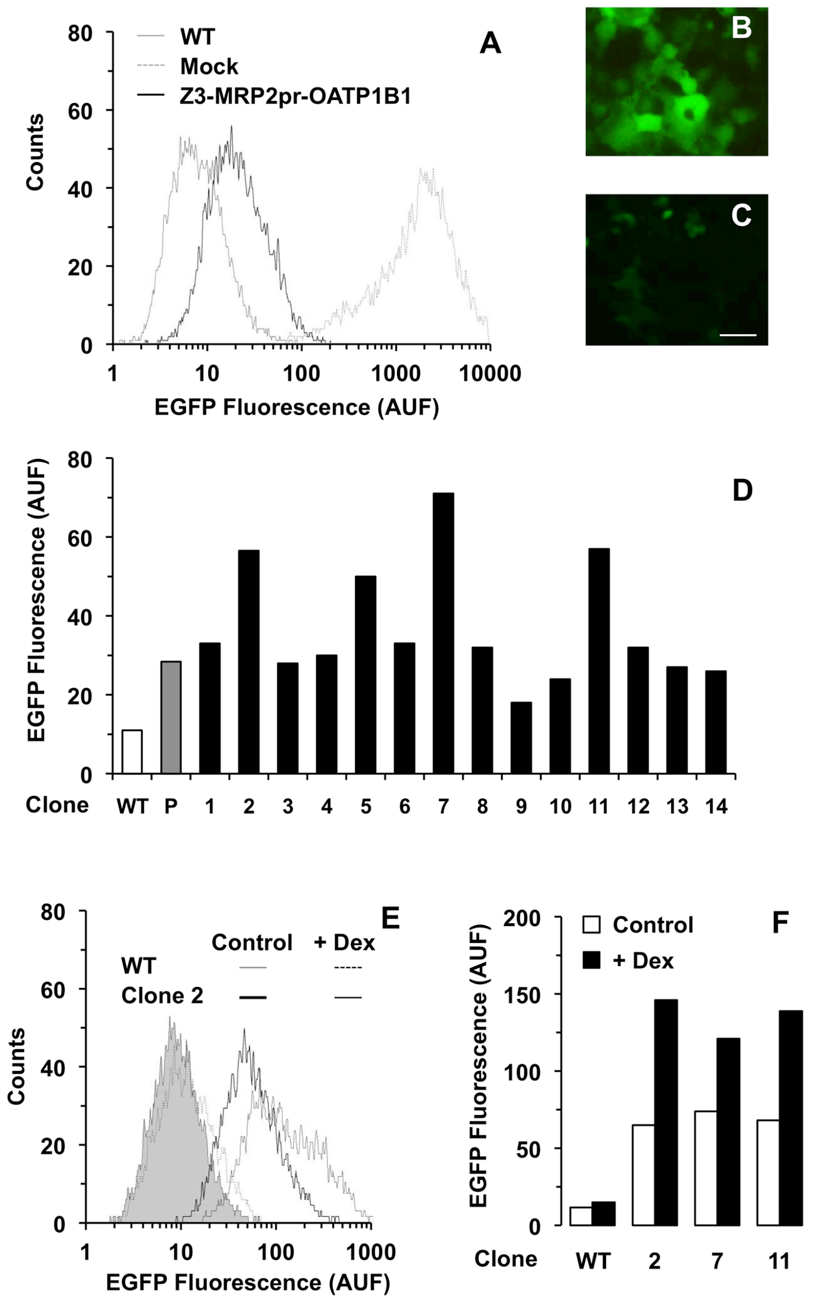

Supplementary Figure 2: (A) Representative frequency histogram of EGFP fluorescence of non-transduced Alexander cells (WT) or cells transduced with an empty lentiviral vector (Mock) or the vector containing Z3-MRP2pr-OATP1B1. Measurements were carried out 4 days after transduction. Representative fluorescence microscopy pictures of Alexander cells transduced with an empty lentiviral vector **(B)** or a vector containing Z3-MRP2pr-OATP1B1 **(C)**. **(D)** EGFP fluorescence of Alexander cells (WT), a polyclonal population (P) of transduced cells and different clones (closed bars). Values are expressed as mean fluorescence measured by flow cytometry. **(E)** Representative frequency histogram of EGFP fluorescence of Alexander cells (WT) and the clone number 2 stably expressing OATP1B1 and EGFP under the control of Z3-MRP2pr, treated with or without (control) 100 nM of Dex for 24 h. **(F)** Mean fluorescence of EGFP in Alexander cells (WT) and clones stably expressing OATP1B1 and EGFP under the control of Z3-MRP2pr, treated with or without (control) 100 nM of dexamethasone (Dex) for 24 h.

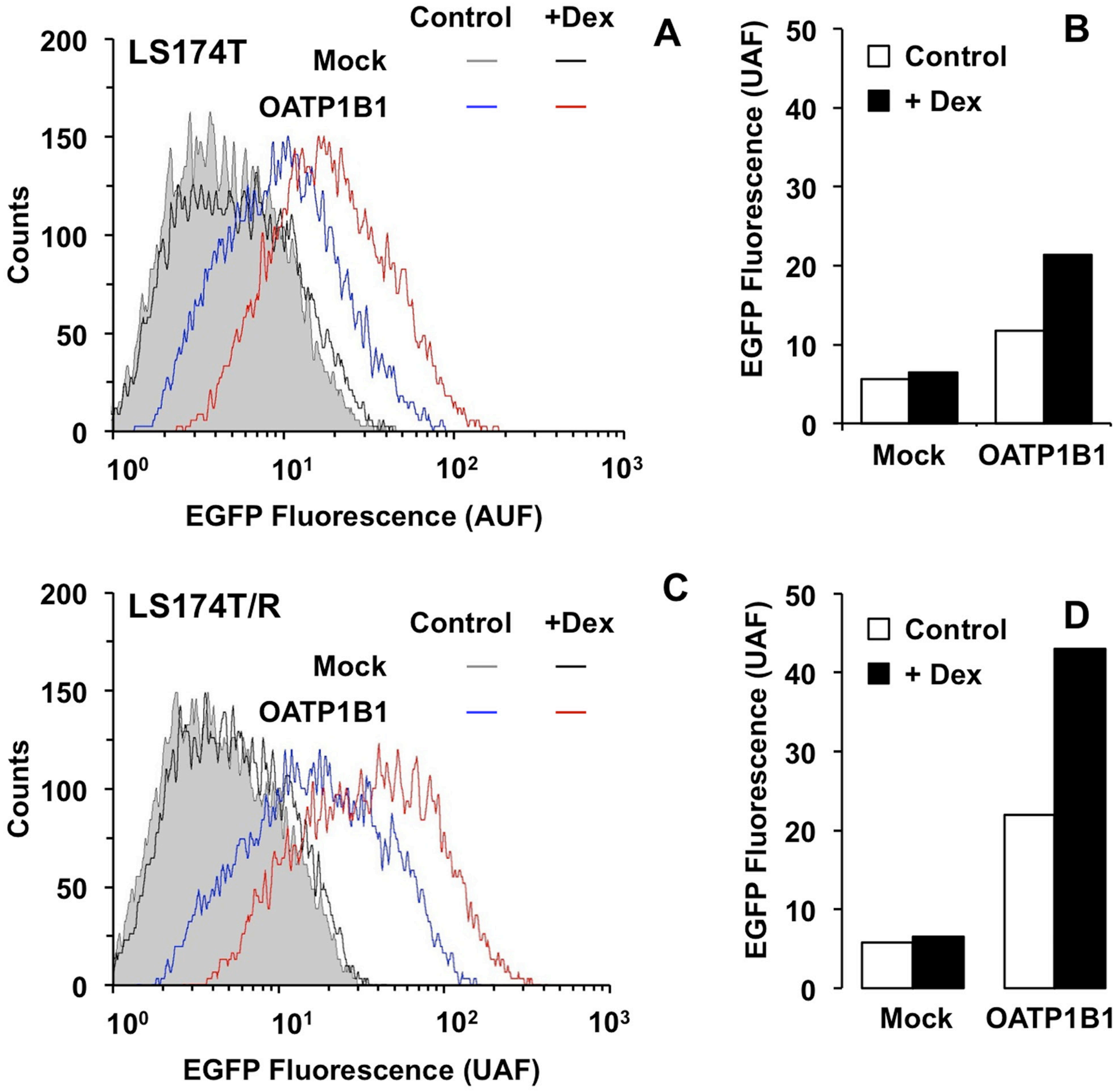

Supplementary Figure 3: Representative frequency histograms of EGFP fluorescence of LS174T (A) and LS174T/R cells (C) transduced with an empty lentiviral vector (Mock) or a vector containing Z3-MRP2pr-OATP1B1 and EGFP, and treated with 100 nM dexamethasone (Dex) for 24 h. Mean fluorescence of EGFP in LS174T **(B)** and LS174T/R **(D)** cells transduced with an empty lentiviral vector (Mock) or a vector containing Z3-MRP2pr-OATP1B1 plus EGFP, and treated with 100 nM Dex for 24 h.

Supplementary Table 1: Oligonucleotide sequence of specific primers used in this study

Application	Sequence (5'-3')	Position (bp)	Type
**Amplification of ORF of OATP1B1**	ATGGACCAAAATCAACATTTG	96	Fw
	ACAATGTGTTTCACTATCTGCCCC	2168	Rv
**QPCR of OATP1B1**	TGCAATGGATTGAAGATGTTCTTGGCA	165	Fw
	CAACCTTTTCCCACTATCTCAGG	580	Rv
**QPCR of GAPDH**	TGAGCCCGCAGCCTCC	91	Fw
	TACGACCAAATCCGTTGACTCC	228	Rv
**Cloning of pWPI-pL**	CAGGTGTCGTGAGGAATTTCGACA		Fw
	CGGAGCCAATTCCCACTCCTTT		Rv

Position of primers is relative to the NM_006446 and NM_002046 sequences for OATP1B1 and GAPDH, respectively. Fw, forward; Rv, reverse.
